# Presentation of Specimens1Reported at the pathological section, Buffalo Academy of Medicine, May 18, 1909

**Published:** 1909-07

**Authors:** J. Henry Dowd

**Affiliations:** Buffalo, N. Y.


### CLINICAL REPORTS.

### Presentation of Specimens[1]

BY J. HENRY DOWD, M. D., Buffalo, N. Y.

PROSTATIC LOBES.

CASE I. In presenting these two specimens which will speak for themselves (specimens shown), I merely wish to call attention to two or three points. It will be noted the three prostatic lobes weigh 26 drams, the weight of the normal prostate being about 6 drams. These were removed by the perineal route nine months ago and in a report from the patient May 12, 1909, he says: I can hold my urine from five to six hours and feel as well as when a boy."

After most careful consideration of this subject, summed up from many perineal and suprapubic sections with observation of the cases of others, the writer would express his conclusions as follows:

1. The mortality in prostatectomy is not high, but in many cases the condition following operation is far worse than it was before; I refer to rectal fistula, permanent incontinence, orchitis with suppuration, and the like.

2. Life, although accompanied by slight inconveniences, is usually far more acceptable than death, especially when the condition can be more or less modified and the distressing symptoms relieved by remedies judiciously employed.

---

1. Reported at the pathological section, Buffalo Academy of Medicine, May 18, 1909

3. Where life is almost a burden, even though the physical condition is bad, and in cases where early operation may prolong life, preventing serious complications, prostatectomy is advisable.

PHOSPHATIC STONES.

Case II. These five stones which are of the phosphatic variety, weight 1980 grains. They were removed by the suprapubic route, a perineal tube being placed in the bladder for drainage. There was an uninterrupted recovery, in three weeks the patient being able to hold his urine four hours, and only an occasional drop appearing at the suprapubic opening.

It is not so much the fact of the perfect result from operation that I would emphasize, but the prevention of recurrence of such a condition.

This patient's urine before operation was alkaline, having a very low phosphatic index. It is well known that alkalinity favors the precipitation of phosphatic crystals; that we rarely find them in acid urine, in the absence of phosphatic calculus of the kidney or bladder. Acidity of urine is due to the acid sodium phosphate; therefore, to change the urine from an alkaline to an acid reaction is the problem.

The treatment is most happily demonstrated by the phosphatic index,—which in this case was very low. During the stay of three weeks in the hospital the patient's neuroinedic condition was carefully attended to. Examination of the urine four weeks after operation showing acid reaction with the phosphatic index gradually rising, and no crystals present.

40 North Pearl Street.

